# Physiological Mechanisms and Associated Pathophysiology of Dysphagia in Older Adults

**DOI:** 10.1177/23337214221142949

**Published:** 2022-12-23

**Authors:** Constantino Estupiñán Artiles, Julie Regan, Claire Donnellan

**Affiliations:** 1 Trinity College Dublin, Ireland

**Keywords:** aging, Alzheimer’s/dementia, biogerontology, frailty, literature review, long-term care, nursing, Parkinson’s disease, stroke

## Abstract

Dysphagia can be a common secondary sequela of neurological and neurodegenerative disorders in older adults. Early screening, identification, and management of dysphagia is essential to avoid serious complications, including malnutrition, dehydration, aspiration pneumonia; and promote quality of life. Although individuals of all ages may experience swallowing difficulties, dysphagia and its complications are more common in older adults. This literature review aims to provide an overview of the physiological mechanisms of normal swallowing in healthy individuals and age-related changes to swallowing function, the pathophysiology of dysphagia associated with three common neurological disorders affecting older adults (stroke, Parkinson’s disease, and dementia), and implications for interdisciplinary clinical practice. Increased awareness of these issues may contribute to a more timely and efficient identification of older adults with dysphagia and to improve overall dysphagia management.

## Introduction

Eating and drinking are basic human needs and, under normal circumstances, a pleasurable experience. A critical component of the eating and drinking process is the act of swallowing, that is, the process of moving food or liquids from the mouth to the stomach safely and efficiently (Smithard, 2018). Although most older adults can swallow effortlessly, difficulty swallowing may develop secondary to neurological or neurodegenerative diseases, head and neck cancers or heart failure, among other causes (Speyer et al., 2022). The medical term dysphagia refers to a difficulty swallowing food and/or fluid or to the sensation that food and/or fluid become obstructed on their transit from the mouth to the stomach (Malagelada et al., 2015). Dysphagia may negatively impact older adults’ physical and psychological well-being as well as social interactions and overall quality of life (Baijens et al., 2016).

Interdisciplinary collaboration is required for effective identification of older adults at risk of dysphagia, assessment of swallowing function and adequate implementation of dysphagia management interventions (Nogueira & Reis, 2013). To this end, a thorough understanding of the anatomy and physiology of swallowing is required by all members of the interdisciplinary team providing direct care to older adults with or at risk of dysphagia. The purpose of this article is to provide an overview of normal swallowing function in healthy older adults including presbyphagia; most common neurological disorders affecting older adults including stroke, Parkinson’s disease, and dementia; and implications for clinical practice.

## Normal Swallow Function in Older Adults

**Figure 1.**
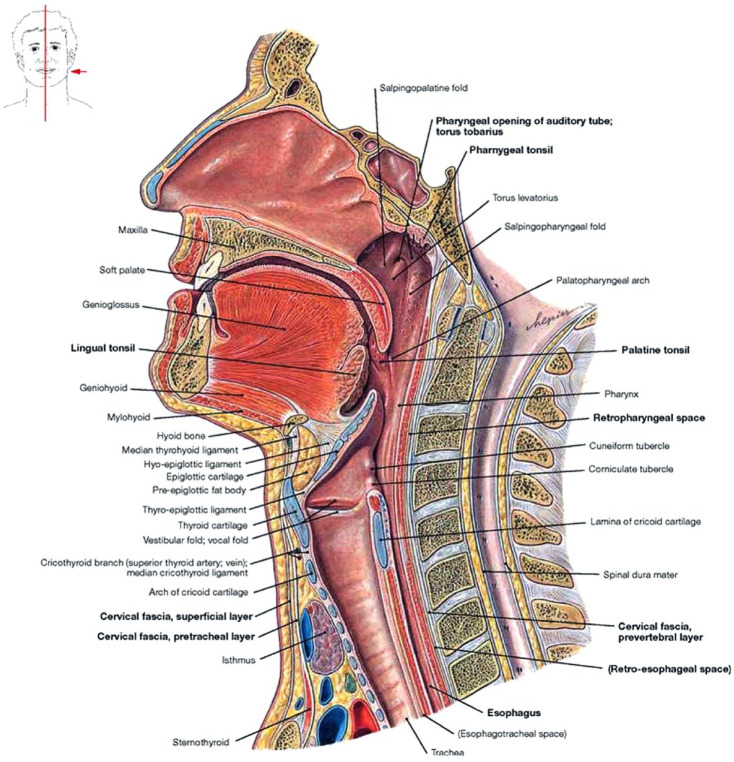
Midsagittal section of the oral cavity, pharynx, and larynx. Sourced with permission from Paulsen (2018).

Respiration and swallowing are two functions performed through the same anatomic pathway. Despite its apparent simplicity, swallowing is a very complex neuromuscular activity that involves an intricated central nervous system process, five cranial nerves (see Table 1) and twenty-six pairs of muscles (see Figure 1) (Baijens et al., 2016; Fregosi & Ludlow, 2014; Simons & Hamdy, 2017).

**Table 1.** Innervation of Cranial Nerves, Structures Involved in Swallowing and Their Role.

Structure	Cranial nerve	Motor function	Sensory function
Temporal muscle, masseter, temporalis, lateral and medial pterigoids, soft palate, larynx, digastric muscle (anterior belly), mylohyoid, submandibular and sublingual salivary glands.	V (Trigeminal)	Secretion of saliva, mastication, bolus formation, lip seal, elevation of the hyoid bone.	Taste, pressure, temperature, and nociceptive stimuli.
Depressors and elevators of the lips, risorius, buccinator, orbicularis oris, tongue, digastric muscle (posterior belly), stylohyoid.	VII (Facial)	Secretion of saliva, bolus formation, lip seal, elevation of the hyoid bone.	Taste, pressure, temperature, and nociceptive stimuli.
Parotid salivary glands, muscles in the pharynx (anterior faucial pillars, palatopharyngeal arch, posterior pharyngeal wall), soft palate, base of the tongue.	IX (Glossopharyngeal)	Controls swallowing movements, parasympathetic control of secretion of saliva.	Taste, pressure, temperature, and nociceptive stimuli.
Cricopharyngeus muscle, pharyngeal constrictors, vocal folds, esophageal muscles, internal laryngeal muscles, palatoglossus, epiglottis, glottis.	X (Vagus)	Fibers innervate the soft palate, pharynx, larynx and esophagus, controls swallowing movements, coughing and voice.	Taste, pressure, temperature, and nociceptive stimuli.
Internal laryngeal muscles, uvular.	XI (Accessory)	Carries motor fibers for the sternocleidomastoid and trapezius muscle, controls swallowing movements, seal of the nasopharynx and vocal folds.	No sensory function.
Intrinsic and extrinsic tongue muscles, geniohyoid, thyrohyoid, omohyoid, sternohyoid, sternothyroid.	XII (Hypoglossal)	Tongue movement, bolus formation, preparing and moving the bolus into the pharynx, seal oral cavity, speech, hyoid bone, and larynx elevation.	No sensory function.

*Source*. Speyer et al. (2022), Auvenshine and Pettit (2020), Sasegbon and Hamdy (2017), Baijens et al. (2016), Royal College of Physicians and British Society of Gastroenterology (2010).

The swallowing process can be divided in three sequential and interconnected stages: oral, pharyngeal, and esophageal stage (Baijens et al., 2016; Sasegbon & Hamdy, 2017). Nevertheless, some authors describe the swallow process in four stages, identifying a previous stage named oral preparatory (Parker & Power, 2013), whereas other authors have identified two substages within the oral stage: the oral preparatory and the propulsive stage (Sasegbon & Hamdy, 2017).

### Oral Stage (Oral Preparatory and Oral Propulsive Stage)

The somatic-voluntary nervous system controls most of the oral stage (Affoo et al., 2013). To swallow liquids, the tongue creates a groove, cupping the liquid by adjusting its shape anteriorly and inferiorly, holding the fluid in preparation for swallowing (Burbidge et al., 2016). Posteriorly, the tongue base elevates forming a barrier that prevents the liquid from spilling into the pharynx before swallow is initiated. With the liquid in position, swallow commences with a drop of the posterior tongue with a nearly simultaneous application of an antero-posterior driving force by the tongue, causing the fluid to flow into the pharynx (Burbidge et al., 2016). These movements of the tongue are achieved through protrudor and retractor muscles, which are innervated by branches of the hypoglossal nerve (XII) (Fregosi & Ludlow, 2014).

For solids, a soft homogeneous mass called a bolus is formed through mastication and by moving the food within the mouth using the tongue and cheeks, mixing it with saliva (Sasegbon & Hamdy, 2017). The tongue controls the food during chewing, forms a bolus and sends information through sensory nerve endings regarding the thickness, volume, temperature, and taste of the food present in the mouth to the swallowing center in the lower brainstem (Logemann et al., 2013). Once the food is ready for swallowing, the tongue pushes it backward with a muscular stripping action against the soft and hard palate (Morris, 2012). Velopharyngeal closure occurs during tongue movement; the soft palate lifts and its side walls constrict to block the nasopharynx, allowing a build-up of pressure in the pharynx while preventing the bolus from moving into the nasopharynx (Pitts, 2014). The pharynx opens and the posterior aspect of the tongue is depressed forming a slide along which the bolus moves due to a wave-like muscular movement from the anterior tongue, entering the pharynx (Sasegbon & Hamdy, 2017).

### Pharyngeal Stage

This stage is believed to be controlled by a sensory recognition center situated in the medulla oblongata in the brainstem (Logemann & Larsen, 2012), with all the events in this phase occurring involuntarily (Burbidge et al., 2016). Sensory information travels from the pharynx to the brainstem through afferent fibers of the V, VII, IX, and X cranial nerves, which terminate in the nucleus tractus solitarius in the medulla oblongata and is then sent to the nucleus ambiguus, which initiates the motor movements of pharyngeal swallow (Zelic et al., 2021). Pharyngeal reconfiguration into a digestive pathway is defined by glossopalatal opening, velopharyngeal closure, hyolaryngeal excursion, and opening of the upper esophageal sphincter (Speyer et al., 2022). Breathing is not possible during this phase due to neural control of breathing in the brainstem and closing of the entrance to the larynx (Zelic et al., 2021). The hyoglossus, geniohyoid, styloglossus, and intrinsic tongue muscles participate co-ordinately to achieve bolus propulsion from the tongue into the hypopharynx. Elevation of the larynx and hyoid bone pull up the laryngeal vestibule behind the epiglottis, helping to make the epiglottis fold over the glottal space and contributes to laryngeal vestibule closure, blocking the entrance to the larynx (Burbidge et al., 2016; Fregosi & Ludlow, 2014; Pitts, 2014).

The hyoid is a small U-shaped sesamoid bone located above the larynx at the height of the third cervical vertebra which consists of the hyoid body, a pair of greater horns and a pair of lesser horns and participates in swallowing, breathing, and speech (Ito et al., 2012). Ten pairs of muscles attach to each side the hyoid bone, divided in suprahyoid muscles and infrahyoid muscles. The geniohyoid, mylohyoid, anterior digastric, hyoglossus, thyrohyoid, and long pharyngeal muscles participate in hyoid bone elevation and both anterior and superior movement of the larynx (Ludlow, 2015). Vocal folds are controlled by the laryngeal muscles, ensuring the airway is closed during swallowing as well as producing a rapid opening and closing to build up pressure for coughing (Ludlow, 2015).

The upper esophageal sphincter comprises three muscles: the cricopharyngeus muscle, the inferior aspect of the inferior pharyngeal constrictor muscle, and the upper portion of the longitudinal esophageal muscle (Regan et al., 2014). The relaxation of the cricopharyngeus muscle along with the upward and forward movement of the larynx caused by the rising of the hyoid bone, makes the opening of the upper esophageal sphincter possible. The V, VII, and IX to XII cranial nerves and axons traveling through the cervical spinal cord (C1–C2) are involved in these actions (Baijens et al., 2016; Fregosi & Ludlow, 2014).

Residue that has not been cleared in time is collected in the pyriform sinuses, which is a pear-shaped structured located in the hypopharynx, posterolaterally to both sides of the laryngeal opening and with their lower aspect immediately above the upper esophageal sphincter (Burbidge et al., 2016). When residue overflows the pyriform sinuses and enters the laryngeal vestibule, a reflexive swallow is triggered by the receptors of the internal branch of the superior laryngeal nerve (Burbidge et al., 2016; Pitts, 2014).

### Esophageal Stage

This stage, also involuntary, commences when the bolus passes into the esophagus through the upper esophageal sphincter (Morris, 2012). The esophagus is a tubular organ measuring 18 to 26 cm long in adults with parasympathetic innervation from lower motor neurons in the nucleus ambiguus (swallowing center) in the brainstem and the vagus nerve and sympathetic innervation from the intermediolateral cell columns of the T1 to T10 spine, whose primary functions are to propel swallowed fluids or food into the stomach and to prevent gastroesophageal reflux (Yazaki & Sifrim, 2012). It is composed by circular muscle layer surrounded by a longitudinal muscle layer, with the proximal esophagus being composed of striated muscle whereas the distal two thirds are composed of smooth muscles. The transition between striated to smooth muscle is gradual over a 4 to 6 cm long segment (Yazaki & Sifrim, 2012).

The base of the tongue initiates the peristaltic movement which clears the pharynx of material and then propagates longitudinally along the esophagus (Royal College of Physicians and British Society of Gastroenterology, 2010). A primary peristaltic wave is initiated in the esophagus and the bolus progresses sequentially with squeezing movements just behind the bolus (Burbidge et al., 2016; Yazaki & Sifrim, 2012). This progressive circular contraction proceeds distally along the esophagus pushing the bolus into the stomach through the relaxed lower esophageal sphincter (Yazaki & Sifrim, 2012) . Simultaneously, the relaxation of the muscles involved in the swallow return the structures in the pharynx to a breathing pathway (Royal College of Physicians and British Society of Gastroenterology, 2010).

## Presbyphagia

Presbyphagia refers to the characteristic age-related changes in head and neck anatomy and in several muscular and neural mechanisms resulting in decreased sensation in the mouth and pharynx and reduced functional reserve (McCoy & Desai, 2018; Namasivayam-MacDonald & Riquelme, 2019). Although the efficiency and safety of the swallow is not compromised, these age-related changes increase the risk of developing dysphagia in older adults with a diminished functional reserve or ability to adapt to health stressors. In the event of an acute illness, the swallowing ability of the older adult may be impaired to an extent in which the swallow becomes unsafe, hence crossing the threshold between presbyphagia and dysphagia (Namasivayam-MacDonald & Riquelme, 2019; Sasegbon & Hamdy, 2017).

Swallowing function in older adults is affected by loss of muscle mass (sarcopenia) in oropharyngeal muscles resulting in reduced tongue pressure, reduced hyolaryngeal elevation, increased duration of upper esophageal sphincter opening and a narrower opening diameter of the upper esophageal sphincter (McCoy & Desai, 2018; Sasegbon & Hamdy, 2017; Sporns et al., 2017). A reduction in the volume of the geniohyoid muscle, which is the muscle that has the most potential to move the hyoid bone anteriorly, has been described in older adults compared to their younger counterparts, resulting in a higher risk of aspiration in older adults (Feng et al., 2014). Also, the hyoid bone elevates up to 2 mm further than required in male older adults, whereas it elevates 8 mm further than required in their younger counterparts, effectively meaning that older males have very little functional reserve and are at a higher risk of swallowing impairment if they become ill (Logemann et al., 2013).

A delay in laryngeal vestibule closure and upper esophageal sphincter relaxation occurs in older adults (McCoy & Desai, 2018; Sasegbon & Hamdy, 2017; Wirth et al., 2016). This is thought to be a consequence of age-related neuronal loss causing impaired sensation, muscle coordination, and brain processing (Sasegbon & Hamdy, 2017). Also, reduced secondary esophageal peristalsis, increased intrabolus pressure and increased impedance to bolus flow have been described in this cohort (McCoy & Desai, 2018). These age-related changes to esophageal motility are rarely symptomatic (Malagelada et al., 2015).

## Dysphagia in Stroke, Parkinson’s Disease, and Dementia

There are two types of dysphagia: oropharyngeal dysphagia, due to malfunction of the oral cavity, pharynx, and upper esophageal sphincter; and esophageal dysphagia, due to malfunction of the esophagus or the esophagogastric junction (Malagelada et al., 2015). Oropharyngeal dysphagia is considered a geriatric syndrome (Baijens et al., 2016). Signs and symptoms of dysphagia include coughing or choking when attempting to swallow food, medication or fluids, unexplained weight loss, painful or effortful swallow, sensation of food sticking on the throat or chest and changes in voice quality following a swallow (Sasegbon & Hamdy, 2017; Wirth et al., 2016). Nevertheless, characteristics of dysphagia may differ depending on its etiology (see Table 2).

**Table 2.** Characteristics of Dysphagia in Stroke, Parkinson’s Disease, and Alzheimer’s Disease and Age-Related Changes to Swallowing Function (Baijens et al., 2016; McCoy & Desai, 2018; Suttrup et al., 2017; Wirth et al., 2016).

Swallowing stage and nerves involved	Presbyphagia	Stroke	Parkinson’s disease	Alzheimer’s disease
Oral StageV—TrigeminalXII—Hypoglossal	Reduced/altered salivary flow Reduced perception of spatial-tactile recognition on tongue and lips Loss of taste Altered lingual pressure Reduced tongue strength Slower initiation of swallow Increased fatigue during eating	Leaking food or fluids from the mouth Tongue incoordination and weakness Oral residue	Inefficient mastication Tongue incoordination and weakness Incoordination of oropharyngeal muscles Oral residue Lingual pumping	Swallowing apraxia Agnosia Changes in dietary habits Delayed initiation of oral stage Refusal to eat or drink Loss of interest in food Prolonged oral stage
Pharyngeal Stage IX—Glossopharyngeal X—Vagus XI—Accessory XII—Hypoglossal	Slower initiation of pharyngeal and laryngeal events Delayed laryngeal vestibule closure Pooling/pocketing in pharyngeal recesses for longer than younger counterparts Increased risk of penetration and aspiration Changes in respiration/swallowing coordination	Delayed initiation of pharyngeal swallow Incoordination and weakness of pharyngeal muscles Prolonged pharyngeal stage Vallecular residue after swallow Residue in pyriform sinuses Diminished cough reflex Penetration and aspiration Silent aspiration	Delayed initiation of pharyngeal swallow Incoordination and weakness of pharyngeal muscles Prolonged pharyngeal stage Vallecular residue after swallow Residue in pyriform sinuses Reduced hyolaryngeal elevation Impaired laryngeal sensation Diminished cough reflex Penetration and aspiration Silent aspiration	Delayed initiation of pharyngeal swallow Prolonged pharyngeal stage Reduced hyolaryngeal elevation Vallecular residue after swallow Residue in pyriform sinuses Penetration and aspiration Silent aspiration
Esophageal Stage X—Vagus Lower motor neurons in the nucleus ambiguus (parasympathetic) Intermediolateral cell columns of the T1–T10 spine (sympathetic)	Reduced pressure drop with upper esophageal sphincter opening Reduced upper esophageal sphincter resting pressure Increased duration of upper esophageal sphincter opening Diminished esophageal sphincter opening Increased impedance to bolus flow Increased intrabolus pressure	Not affected	Weakened esophageal peristalsis Increased intrabolus pressure Distal esophageal spasms Reflux	Not affected

Dysphagia may lead to malnutrition and dehydration due to impaired deglutition efficacy and aspiration and subsequent respiratory tract infection and aspiration pneumonia due to impaired swallowing safety (Rofes et al., 2011). Aspiration occurs when foreign material passes beyond the vocal folds, whereas if it remains above the glottis level, it is called penetration. A reflex cough in response to material threatening to or entering the airway occurs naturally in healthy individuals (Morris, 2012). However, laryngeal penetration and aspiration may happen without coughing, which is known as silent aspiration (Baijens et al., 2016). The aspiration of large quantities of food, fluids, or gastric content may result in aspiration pneumonitis, which is characterized by a sudden onset that may resolve rapidly unless an infection develops (Campbell-Taylor, 2008). Aspiration pneumonia has been defined as a respiratory infection caused by the inhalation of foreign substances contaminated with pathogenic microorganisms (Wirth et al., 2016). This is a serious complication and is currently considered one of the leading causes of death in older adults with dysphagia secondary to dementia and Parkinson’s disease (Baijens et al., 2016; Londos et al., 2013; Schiffer & Kendall, 2019).

### Dysphagia in Stroke

Current evidence suggests that swallowing is mediated by multiple distinct cortical and subcortical regions and that dysphagia may develop following a lesion to primary and secondary somatosensory and motor cortices, inferior frontal gyrus, supramarginal gyrus, supplementary motor area, anterior cingulate cortex, orbitofrontal cortex, the insula, and operculum (Cheng et al., 2022). Stroke related dysphagia may be caused by a loss of functional connectivity in the swallowing network resulting in a decreased activation in both the affected and contralateral hemisphere (Wirth et al., 2016). The effects of stroke are various including disruption to both the oral and pharyngeal stages of swallowing due to the inability to retain food or fluids in the mouth or incoordination or weakness of the tongue or the pharynx (Wirth et al., 2016). Although hemispheric specialization for the different stages of swallowing has been reported, with the left hemisphere involved mostly on processing the oral phase and the right hemisphere being more active during the pharyngeal phase (Teismann et al., 2011; Wirth et al., 2016), the relationship between stroke location and characteristics of dysphagia remains poorly defined (Cheng et al., 2022).

Damage to the dominant cerebral hemisphere has been associated with a higher risk of dysphagia and aspiration (Sasegbon & Hamdy, 2017). Other authors report that right hemispheric stroke is associated with oropharyngeal dysphagia, whereas left hemispheric lesions are associated with oral stage dysfunction (Cheng et al., 2022; Sporns et al., 2017). Some authors have reported that lesion to the right hemisphere result in more severe dysphagia with significant pharyngeal dysmotility and reduced hyolaryngeal elevation, increased pharyngeal residue and prolonged pharyngeal events, resulting in a higher risk of aspiration (Cheng et al., 2022). May et al. (2017) and Wilmskoetter et al. (2018) found that stroke patients showed impaired oral and/or pharyngeal swallowing mechanics irrespective of whether the stroke affected the right or left hemisphere, although these impairments were significantly more severe in patients with right-sided lesions. Difficulties in bolus preparation and transport, spillage, delayed swallowing response, pharyngeal residue, weak pharyngeal constriction, and reduced hyolaryngeal excursion have been described in patients during the subacute phase with both left and right ischemic stroke (Teismann et al., 2011). More recently, Fernández-Pombo et al. (2019) did not find statistically significant associations between location or type of the stroke and presence of dysphagia or its characteristics. Interestingly, a statistically significant association between the volume of the lesion and alterations in the safety and efficacy of swallowing in patients with larger lesions was reported. However, other authors have not found an association between lesion size and severity of dysphagia (Cheng et al., 2022).

Although most acute stroke patients will fully recover their swallowing ability spontaneously within the first few days, around 11% of stroke patients will continue to have dysphagia at 6 months (Cohen et al., 2016). Vilardell et al. (2017) found that impaired swallowing in chronic stroke patients is mainly caused by a weak tongue propulsion force and delayed laryngeal vestibule closure.

### Dysphagia in Parkinson’s Disease

Idiopathic Parkinson’s disease is known to affect the oral and pharyngeal stages of swallowing even at early stages, although severe dysphagia is often observed in advanced stages (Nascimento et al., 2020; Suttrup & Warnecke, 2016). Inefficient mastication, prolonged pharyngeal stage, impaired relaxation of the upper esophageal sphincter, reduced elevation of the hyoid and larynx, reduced posterior tongue movement and uncoordinated or reduced contraction of oropharyngeal muscles causes an increased amount of pharyngeal residue which, in addition to impaired laryngeal sensation and cough response, may lead to silent aspiration (Argolo et al., 2015; Hammer et al., 2013; Sasegbon & Hamdy, 2017). Nascimento et al. (2020) found that impaired swallow efficacy in individuals with Parkinson’s disease is volume-dependant, with more individuals showing signs of swallowing impairment, especially substantial amounts of oral and pharyngeal residue, with larger boluses. Individuals with Parkinson’s disease present with higher airway somatosensory detection thresholds, which may lead them to believe they have swallowed the entire bolus when that is not the case, further contributing to oropharyngeal residue (Hammer et al., 2013). Lingual pumping, a repetitive, involuntary, anteroposterior tongue movement on the soft palate that occurs prior to transferring the bolus to the pharynx commonly observed in Parkinson’s disease patients, is associated with incoordination during the oral stage, pharyngeal residue, and aspiration (Argolo et al., 2015).

Due to autonomic dysfunction, the esophageal stage of swallowing is also affected in idiopathic Parkinson’s disease. Hypotensive esophageal peristalsis and increased intrabolus pressure may occur at any stage of the disease, nevertheless, they are more common and present more severely at advanced stages of the disease (Suttrup et al., 2017). Esophageal motility impairment may lead to distal esophageal spasms in some patients, although esophageal spasms can be present in early stages of the disease as hypoperistalsis or aperistalsis (Suttrup et al., 2017).

### Dysphagia in Dementia

Dysphagia in Alzheimer’s disease is believed to be caused by functional changes to the cortical swallowing network and dysfunction of the autonomous nervous system affecting the oral and pharyngeal stages of swallowing (Affoo et al., 2013). Older adults with Alzheimer’s disease usually need direct assistance with eating and drinking due to swallowing apraxia, a discoordination of lingual, labial, and mandibular functioning during the oral stage, and agnosia (Wirth et al., 2016). This inability to recognize the bolus as food results in delayed initiation of the oral stage, prolonged oral transit times, and delayed pharyngeal initiation of swallow. In addition, reduced hyolaryngeal excursion, increased pharyngeal residue and penetration/aspiration are often observed in individuals with Alzheimer’s disease and other dementias (Affoo et al., 2013; Wirth et al., 2016; Zelic et al., 2021). It seems that eating and swallowing difficulties in individuals with Alzheimer’s disease are less severe than in individuals with frontotemporal lobe dementia and Lewy body dementia, although they may develop earlier in Alzheimer’s (Affoo et al., 2013).

Patients with dementia with Lewy bodies may present with similar motor symptoms and oropharyngeal muscle dysfunction as observed in individuals with Parkinson’s disease (Londos et al., 2013; Sasegbon & Hamdy, 2017). Prolonged pharyngeal stage, pharyngeal residue, and penetration/aspiration is often observed in this cohort (Londos et al., 2013).

## Implications for Clinical Practice

Clinicians involved in the care the older adults need a clear understanding of changes in swallowing function due to aging to distinguish between normal swallow changes in older adults and dysphagia to successfully deliver appropriate treatment and management interventions. This will avoid both implementing unnecessary compensatory strategies that may lead to restrictions in nutritional intake and reduced quality of life, and undermanaging older adults with dysphagia, which may lead to dehydration, malnutrition, and aspiration (McCoy & Desai, 2018). Nurses, as they provide around the clock care at the bedside, are usually the healthcare professionals that may first observe signs of swallowing difficulties in patients, for example at mealtimes. Local protocols may indicate the administration of swallow screening tests routinely or in cases in which dysphagia is suspected. Although these tests may be administered by any member of the interdisciplinary team, they are usually administered by nurses (Estupiñán Artiles et al., 2021). Swallow screening constitutes the first step in the dysphagia management plan and is defined as a pass or fail procedure to identify individuals who are at risk of dysphagia and require further evaluation (Speyer et al., 2022). Swallow screening protocols have been developed for early identification of dysphagia in acute stroke patients (Wirth et al., 2016), Parkinson’s disease (Burgos et al., 2018), and older adults admitted for long-term care (Park et al., 2015). These protocols may require that patients remain “nil per oral” if dysphagia is suspected until a comprehensive assessment of swallowing function has been conducted.

Older adults presenting with signs and symptoms of dysphagia, or that fail a swallow screening test, should be referred to the speech and language therapist for assessment of swallowing function, which may include instrumental assessment, and management recommendations (Baijens et al., 2016). Dysphagia management strategies include both compensatory strategies, such as food and fluids modifications and postural changes, and rehabilitation interventions (Baijens et al., 2016; Wirth et al., 2016). Because modified diets provide less calories than unmodified diets and dysphagia increases the risk of malnutrition in the older adult, nutritional supplementation, extra snacks, and food fortification should be considered (Miles et al., 2014; Morris, 2012). Therefore, older adults with dysphagia, particularly those that have been recommended to follow a modified diet, should be referred to a dietitian for individualized dietary advice, nutritional support, and education (Miles et al., 2014).

Importantly, polypharmacy is common in older adults and many drugs may negatively impact swallowing function in this cohort (Baijens et al., 2016; Miles et al., 2014). Physicians and pharmacists have a critical role in reviewing older adults’ medication and its possible effects in swallowing function as well as in prescribing alternative formulations for those patients who cannot swallow their medication safely (Miles et al., 2014).

Dysphagia in the older adult is multifactorial and is associated with multiple comorbidities (Baijens et al., 2016). Consequently, there is no standard approach to managing dysphagia in older adults; instead, individualized care plans and goals should be implemented to address care needs (McCoy & Desai, 2018).

## Conclusion

Although several changes to anatomical structures and physiological processes involved in swallowing function occur due to aging, dysphagia is not a consequence of old age. In older adults, dysphagia usually develops secondary to neurological and neurodegenerative disorders. A comprehensive understanding of what constitutes a normal swallow in older adults is of paramount importance for accurately distinguishing between age-related changes to swallowing function and abnormal swallowing. Coordinated involvement of an interdisciplinary team consisting of speech and language therapists, dieticians, nurses, pharmacists, and physicians, at a minimum, is required for timely identification older adults with or at risk of dysphagia, appropriate management interventions, minimizing dysphagia related complications and optimizing quality of life.
